# Metastatic Calcinosis of Aortic Valve Secondary to Renal Failure Mimicking Infective Endocarditis

**DOI:** 10.1155/2016/3916507

**Published:** 2016-09-25

**Authors:** Noman Ahmed Jang Khan, Masroor A. Khan, Guillermo Juan Morell Chardon

**Affiliations:** ^1^Temple University/Conemaugh Memorial Hospital, Johnstown, PA, USA; ^2^Interventional Cardiology, Houston Methodist Hospital, Houston, TX, USA; ^3^University of Texas, Houston, TX, USA

## Abstract

End stage renal disease has a list of consequences, cardiovascular being the most common. Inefficient dialysis can cause significant deposition of calcium all over the body, including heart valves making heart function impaired. We illustrate a case of 38-year-old female with end stage renal disease on peritoneal dialysis. The patient had been complaining of pain and swelling of the right hand for the last few months and had been seen by hand surgeon and was admitted electively for the biopsy of hand lesions. Before her planned surgery, she developed severe shortness of breath. Urgent echocardiogram revealed severe aortic regurgitation and large vegetation on the aortic valve. Infective endocarditis was suspected but blood cultures were negative for any microorganism and the patient did not meet the Duke criteria. Because of her hemodynamic instability immediate mechanical valve replacement surgery was performed. The pathology report showed extensive calcification and myxoid degeneration. No infectious agent was found. Later on, biopsy of her hand lesions showed extensive calcification with macrophages and giant cells. No atypia or malignancy was identified. This is a rare case of the metastatic calcinosis of aortic valve secondary to renal failure mimicking aortic valve infective endocarditis.

## 1. Case History

**Figure 1:**
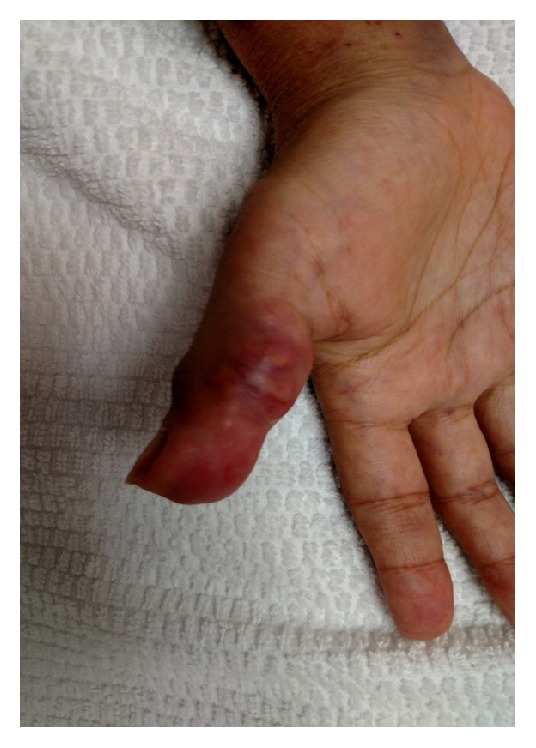
Right thumb lesions.

**Figure 2:**
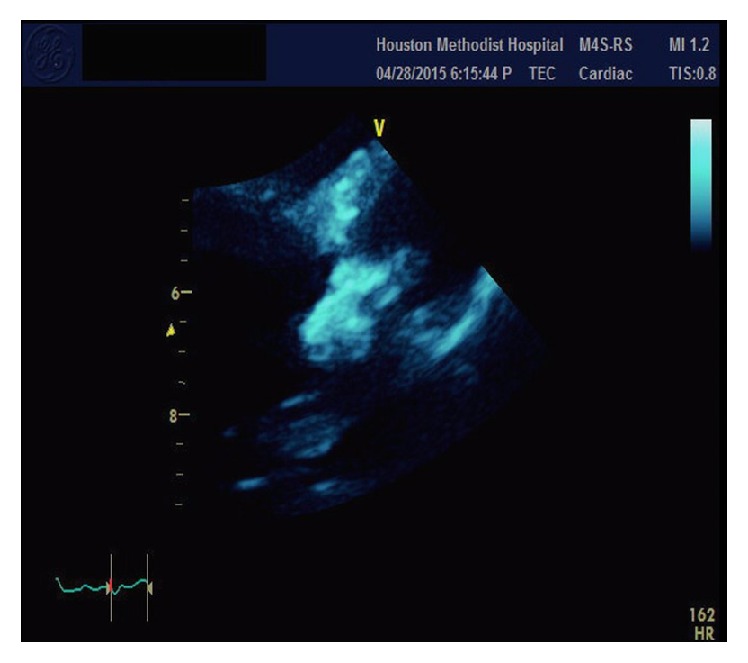
Aortic valve vegetation in parasternal long axis view.

**Figure 3:**
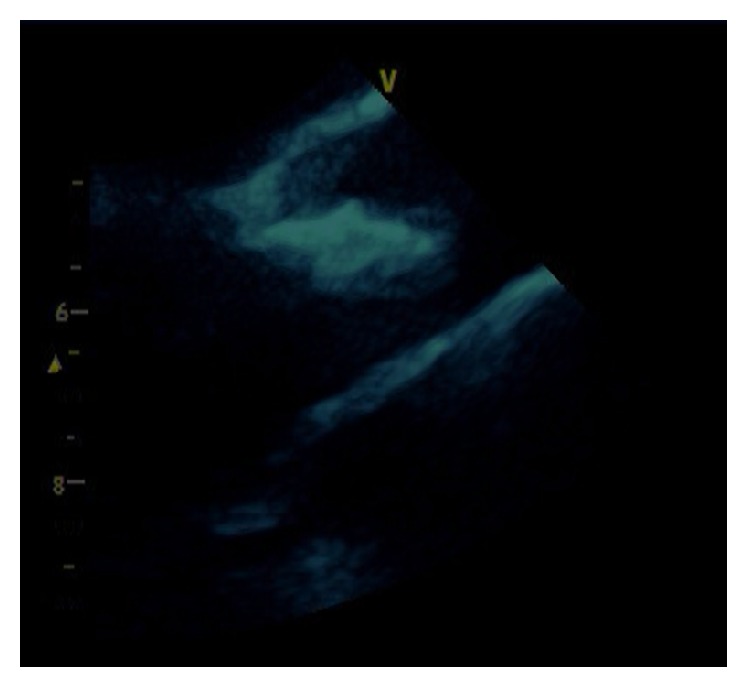
Aortic valve vegetation in parasternal view.

**Figure 4:**
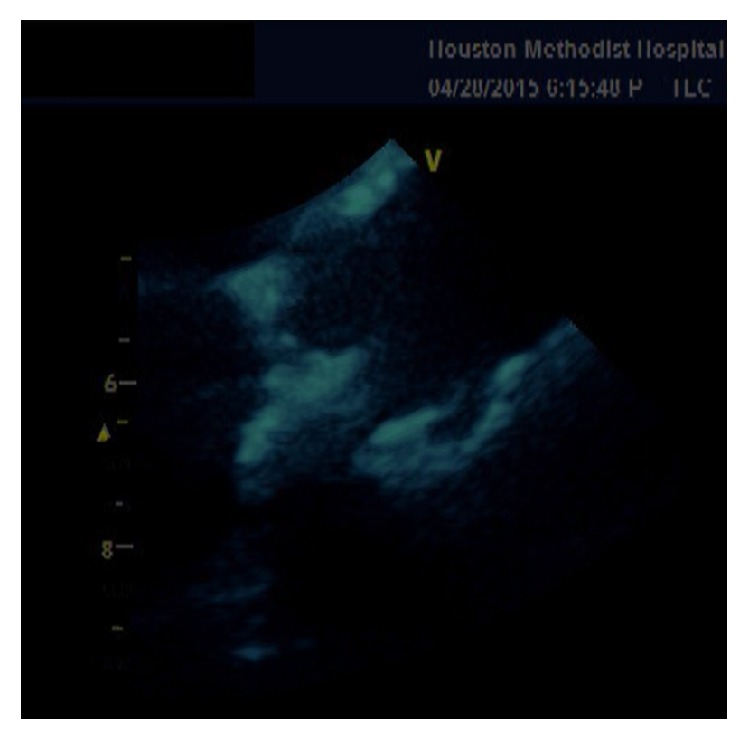


A 38-year-old female with past medical history of end stage renal disease secondary to IgA nephropathy on peritoneal dialysis, hypothyroidism, gout, hypertension, and peritonitis presented with painful swellings of both hands more prominent on the right thumb (Figure 1). During the few months before her presentation her calcium was in the range of 7.7 to 9.7 mg/dL and phosphorus in the range of 8.9 to 15.8 mg/dL. CT scans of the abdomen and pelvis revealed stone in the right kidney and soft tissue calcification in the hip. Aspiration of the thumb swellings at the time of presentation ruled out gout and showed diffuse deposition of calcium phosphate. X-rays of her hand showed diffuse calcification in the volar aspect of right thumb. She was planned for the removal of these growths under general anesthesia, but one day prior to her procedure she developed severe shortness of breath. Urgent echocardiogram was done which showed severe aortic regurgitation and large vegetation on the aortic valve (Figures 2, 3, and 4). The patient was hemodynamically unstable and therefore urgent surgery for mechanical aortic valve replacement was performed. Pathology report revealed irregular soft tissue calcification on cut surface. Microscopically the specimen was composed of myxoid degeneration with calcification and areas of acute and chronic inflammation. No infectious agent was found. Surgery for her hand lesions was performed after her acute condition settled down. A biopsy was taken and histopathology revealed extensive calcification with macrophages and giant cells. No atypia or malignancy was found.

This is a very rare case of metastatic calcinosis secondary to end stage renal disease affecting aortic valve mimicking infective endocarditis.

## 2. Case Discussion

Cardiovascular complications are the most common cause of death in patients with end stage renal disease on dialysis. Left ventricular hypertrophy, coronary artery disease, arrhythmias, myocardial fibrosis, and heart failure are the major cardiovascular complications in renal failure [1]. Valve calcification is a rare sequel of renal failure. Mitral valve is the most common valve affected and it usually manifests as mitral annular calcification (MAC), with aortic valve being the second most common [2]. In rare cases as in our patient a tumor like calcified mass can also arise close to the mitral valve which makes it difficult to distinguish from vegetation [3]. Impaired calcium phosphate metabolism is considered to be the main factor in the pathogenesis of valve calcification. Phosphate levels above 6.5 mg/dL and calcium phosphate product above 72 mg^2^/dL^2^ are associated with increased risk of valvular calcification [4]. Other molecules implicated in the pathogenesis are Osteoprotegerin, RANK, RANKL, Fetuin A mineral complexes, and FGF-23/klotho complexes [5]. In our patient phosphate was as high as 15.8 mg/dL and calcium phosphate product was 140.62 mg^2^/dL^2^. The use of vitamin D for secondary hyperparathyroidism in renal failure and calcium containing phosphate binders is associated with net positive calcium balance causing significant contribution in valvular calcification [6].

Aortic valve calcification can present as stenosis, regurgitation, or severe insufficiency as in our case. Unlike our patient who presented abruptly with severe aortic regurgitation, usual calcinosis causes chronic progressive regurgitation. Aortic valve calcification can be diagnosed by echocardiography or electron beam computed tomography.

The management of valvular calcification in end stage renal disease is a challenge. Prevention is the primary goal of therapy. The importance of calcium and phosphate levels is widely addressed. Dietary phosphate restriction is effective in preventing valvular calcification. Increased dialysis can effectively remove excess phosphorus from the body ameliorating the catastrophe, but nearly all patients require phosphate binders to control phosphate levels. The traditional aluminum and calcium containing phosphate binders are largely replaced by newer agents because of aluminum intoxication and hypercalcemia, respectively. Sevelamer hydrochloride, an iron binding resin has appeared to be effective in reducing phosphate levels [7]. Our patient was on peritoneal dialysis and was noted to have increased phosphate levels up to 15 mg/deal during the last several months before her presentation to the hospital. Increased phosphate and secondary hyperparathyroidism might have put forth extensive metastatic calcification of her aortic valve and caused severe aortic insufficiency for which she needed mechanical aortic valve replacement. This is a very rare complication of renal failure causing significant morbidity and mortality in renal failure patients.

## Supplementary Material

The metastatic calcification of the aortic valve with severe aortic valve insufficiency can be clearly appreciated on the trans thoracic echocardiogram.

## Additional Points

End stage renal disease hampers the electrolyte balance, making patients susceptible to complications such as metastatic calcification. These calcifications might mimic features of infective endocarditis making diagnosis and management quite difficult.

Our case highlights the devastating aspects of metastatic calcification of aortic valve secondary to end stage renal disease. Efficient dialysis to prevent the unwanted rise of calcium and phosphate is very important. To avoid complications such as metastatic calcification close monitoring of calcium and phosphate is helpful.
